# Functional CRISPR screen identifies AP1-associated enhancer regulating FOXF1 to modulate oncogene-induced senescence

**DOI:** 10.1186/s13059-018-1494-1

**Published:** 2018-08-17

**Authors:** Ruiqi Han, Li Li, Alejandro Piñeiro Ugalde, Arieh Tal, Zohar Manber, Eric Pinto Barbera, Veronica Della Chiara, Ran Elkon, Reuven Agami

**Affiliations:** 1 Division of Oncogenomics, The Netherlands Cancer Institute, Plesmanlaan 121, 1066 CX Amsterdam, The Netherlands; 2 Department of Genetics, Erasmus University Medical Center, Wytemaweg 80, 3015 CN Rotterdam, The Netherlands; 3 Oncode Institute, Amsterdam, The Netherlands; 4 Department of Human Molecular Genetics and Biochemistry, Sackler School of Medicine, Tel Aviv University, 69978 Tel Aviv, Israel

**Keywords:** CRISPR, Functional screen, Enhancers, Oncogene-induced senescence, Gene regulation, AP1, FOS, JUN, FOXF1

## Abstract

**Background:**

Functional characterization of non-coding elements in the human genome is a major genomic challenge and the maturation of genome-editing technologies is revolutionizing our ability to achieve this task. Oncogene-induced senescence, a cellular state of irreversible proliferation arrest that is enforced following excessive oncogenic activity, is a major barrier against cancer transformation; therefore, bypassing oncogene-induced senescence is a critical step in tumorigenesis. Here, we aim at further identification of enhancer elements that are required for the establishment of this state.

**Results:**

We first apply genome-wide profiling of enhancer-RNAs (eRNAs) to systematically identify enhancers that are activated upon oncogenic stress. DNA motif analysis of these enhancers indicates AP-1 as a major regulator of the transcriptional program induced by oncogene-induced senescence. We thus constructed a CRISPR-Cas9 sgRNA library designed to target senescence-induced enhancers that are putatively regulated by AP-1 and used it in a functional screen. We identify a critical enhancer that we name *Enh*^*AP1-OIS1*^ and validate that mutating the AP-1 binding site within this element results in oncogene-induced senescence bypass. Furthermore, we identify FOXF1 as the gene regulated by this enhancer and demonstrate that FOXF1 mediates *Enh*^*AP1-OIS1*^ effect on the senescence phenotype.

**Conclusions:**

Our study elucidates a novel cascade mediated by AP-1 and FOXF1 that regulates oncogene-induced senescence and further demonstrates the power of CRISPR-based functional genomic screens in deciphering the function of non-coding regulatory elements in the genome.

**Electronic supplementary material:**

The online version of this article (10.1186/s13059-018-1494-1) contains supplementary material, which is available to authorized users.

## Background

Over the last decade, large-scale genomic projects identified hundreds of thousands of regulatory elements (REs) in the human genome; most of them are putative enhancers [1, 2]. Identification of candidate enhancer regions was mainly based on profiling of characteristic histone modifications (e.g. H3K27ac and H3K4me1) and binding of transcriptional activators (e.g. p300). Recently, enhancer-RNA (eRNA) expression, typically transcribed bi-directionally at promoter-distal cis-REs, was indicated as a sharp feature of active enhancers and was utilized for the systematic discovery of enhancers across the genome [3]. Importantly, changes in eRNA production correlate with changes in the enhancer activity [4, 5]. Yet, functional characterization of the plethora of candidate enhancer elements is a major genomic challenge [6]. High-throughput reporter assays to probe the functions of regulatory regions were developed in recent years [7]. However, these methods separate putative REs from their native chromosome, so that any effect of chromatin context and long range regulatory interactions is lost. Furthermore, definitive demonstration of the function of the RE requires their perturbation in situ. The maturation of novel genome-editing technologies is revolutionizing our ability to interrogate the function of the non-coding genome. This potential was demonstrated by pioneering CRISPR-based functional genomic screens that systematically targeted non-coding elements in the human genome [8–11].

In one of these CRISPR-based functional genomic screens, we focused on oncogene-induced senescence (OIS), which is a cellular state of irreversible proliferation arrest that is enforced in face of excessive oncogenic activity (oncogenic stress) [8]. OIS is a major barrier against cancer transformation [12, 13]; therefore, overcoming OIS is a critical step in tumorigenesis [14]. Activation of this process is largely dependent on p53 [13, 15]; consequently, its bypass by cancer cells is mainly achieved by emergence of somatic mutations (SM) in p53 or other components of its pathway [16, 17]. As p53 is an enhancer-binding transcription factor (TF), whose function in transcriptional regulation is required for its tumor suppressive activity, we previously performed a CRISPR-based functional genomic screen that systematically targeted p53-bound enhancers [18]. That screen uncovered several p53-bound REs that are required for the activation of OIS. However, aberrant expression of oncogenes can lead to the activation of additional TFs whose function is also critical for the establishment and/or maintenance of OIS. In the current study, we aimed at the identification of such TFs and discovery of additional enhancers that are required for the establishment of OIS. We carried out an unbiased profiling of enhancers activated upon oncogenic stress, which indicated AP-1 as a major regulator of the transcriptional program induced by OIS. We thus generated a CRISPR-Cas9 single guide RNA (sgRNA) library designed to target enhancers putatively regulated by AP-1 and used it in a functional screen to identify those required for OIS. This screen detected *Enh*^*AP1-OIS1*^, an AP-1 bound enhancer that is hyper-activated in OIS and whose abrogation results in OIS bypass. Furthermore, we identified *FOXF1* as the target gene of this enhancer and demonstrated that it regulates the senescence phenotype.

## Results

### Genome-wide identification of OIS-induced enhancers

**Fig. 1:**
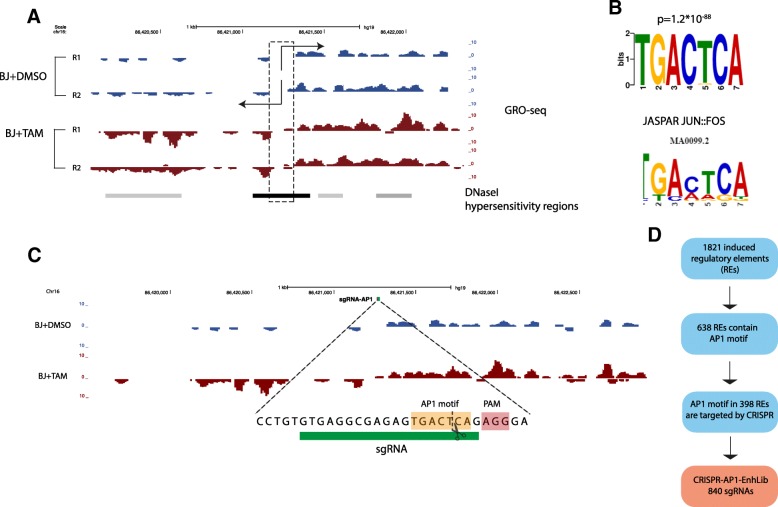
Design of a CRISPR screen targeting AP1 enhancers which are activated upon oncogenic stress. **a** An example of an enhancer whose activity is induced in response to oncogenic stress. Enhancer activity is inferred from the typical bi-directional transcription of eRNAs (BJ + DMSO indicates proliferating cells and BJ + 4-OHT indicates senescent cells); genomic regions that show DNase hypersensitivity (DHS), as determined by ENCODE, are shown by the *gray track*). Overall, our GRO-seq analysis identified 1821 regulatory elements (REs; enhancers or promoters) whose activity was induced in BJ cells in face of RAS activation. **b** De novo motif analysis detected highly significant enrichment of the FOS:JUN (AP1) DNA motif in the REs that were induced upon oncogenic stress. *Top*: the enriched motif detected in our dataset; *bottom*: the AP1 motif from the JASPAR DB [49]. **c** An example for occurrence of an AP1 motif within an enhancer that was induced upon oncogenic stress, that is located close enough to an NGG PAM motif, resulting in Cas9-mediated DNA cleavage that occur within the motif (Cas9 cleavage occurs ~ 3 nt before the PAM). Overall, we identified 398 induced REs with AP1 motif that met this requirement (Cas9 cleavage within a margin of 5 nt with respect to the motif). **d** Statistical summary of the *CRISPR-AP1-EnhLib* used in our functional screen

We previously carried out a CRISPR-based screen aimed at identification of p53-bound enhancers that are required for OIS [17]. That screen was confined to regions that are directly bound by p53 as detected by p53 chromatin immunoprecipitation-sequencing (ChIP-seq) analysis and therefore missed enhancers that are critical for OIS enforcement and are regulated by other TFs, in either a p53-dependent or -independent manner. To overcome this limitation and to globally screen DNA REs activated by OIS without being biased by preselection of a candidate TF, we first sought to comprehensively detect all the enhancers that are activated upon oncogenic stress. To this goal, we utilized Global Run-On sequencing (GRO-seq), a nascent RNA detection method [19], that allow robust determination of eRNA expression, as a quantitative measure for enhancer activity [7, 8, 18]. We used a cellular system in which the oncogene RAS^G12V^ was induced in hTERT-immortalized BJ cells (BJ-indRAS^G12V^). As these cells contain wild-type p53, oncogenic stress results in a very potent activation of OIS and proliferation arrest [20]. Exploiting bi-directional transcription as a hallmark of transcriptional activity at enhancers and promoters [7, 21, 22], we detected 1821 REs whose activity was induced in BJ-indRAS^G12V^ upon oncogene induction for 14 days (Fig. 1a; Additional file 1: Table S1). Next, we bioinformatically searched for TFs that potentially mediate the activation of these regions by performing de novo DNA motif enrichment analysis. Remarkably, we found that the regulatory regions activated during the induction of OIS were significantly enriched for the binding motif of the AP1 (FOS:JUN) TF (Fig. 1b). Overall, these results suggest an important role for AP1 in the regulation of the transcriptional response to oncogenic stress.

Therefore, we constructed a CRISPR library that systematically targets OIS-induced DNA elements that are putatively regulated by AP1 and performed a functional genetic screen to identify those that are required for OIS activation. The de novo motif analysis detected 762 AP1 motifs in 638 OIS-induced REs (over-representation *p* value = 1.2*10^−88^). We examined which of these motif occurrences can be targeted by CRISPR-Cas9, given the requirement for the presence of the NGG PAM motif near the AP-1 motif. WE required that the Cas9-madiated DNA cut will occur either within the motif itself or up to a margin of 5 nt with respect to it (Fig. 1c). Of the 638 OIS-induced REs containing AP1 motif, 398 (62%) met this criterion, with most motifs targeted by 2–3 distinct sgRNAs. Accordingly, we designed 840 sgRNAs that target AP1 motifs in 398 OIS-induced REs. We cloned these sgRNAs as a pool into pLentiCRISPRv2 vector and generated a plasmid library referred herein as *CRISPR-AP1-EnhLib* (Fig. 1d and Additional file 1: Table S2).

### CRISPR screen targeting OIS-induced enhancers with AP-1 motif

**Fig. 2:**
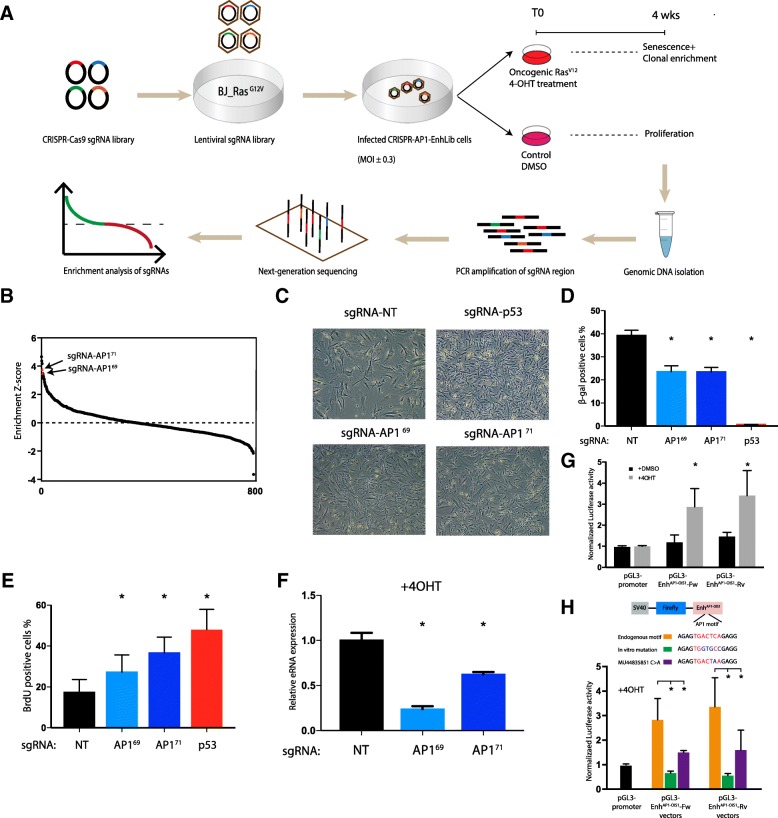
Functional CRISPR screen discovers a novel enhancer required for OIS. **a** *Schematic representation* of the set-up of our functional screen. **b** Results of the CRISPR screen. sgRNAs are sorted by the enrichment score based on the ratio between their prevalence in the BJ + 4-OHT and BJ + DMSO control populations (measured 4 weeks after 4-OHT treatment). *Y-axis* shows Z scores of the mean sgRNA enrichment scores (calculated over the four replicates of the screen). Colored in *red* are two sgRNAs, sgRNA-AP1^69^ and sgRNA-AP1^71^, that target the same enhancer, called here *Enh*^*AP1-OIS1*^ **c** Individual transductions of sgRNA- AP1^69^ and sgRNA-AP1^71^ validated that they cause OIS bypass. sgRNA targeting p53 was used as a positive control and a non-targeting (NT) sgRNA was used as a negative control. **d** Targeting *Enh*^*AP1-OIS1*^ by either sgRNA-AP1^69^ or sgRNA-AP1^71^ caused OIS bypass as measured by β-gal staining, a canonical mark for senescence (p53ko used as a positive control). Data shown represent mean (SD), *n* = 4. *p < 0.05. **e** Targeting *Enh*^*AP1-OIS1*^ by either sgRNA-AP1^69^ or sgRNA-AP1^71^ resulted in enhanced proliferation as measured by BrdU staining (p53ko used as a positive control). Data shown represent mean (SD), *n* = 4. *p < 0.05. **f** Measurement of eRNA production at *Enh*^*AP1-OIS1*^ in cells with the indicated sgRNAs. eRNA levels are significantly decreased upon mutagenesis of the AP1 binding site caused by either sgRNA-AP1^69^ or sgRNA-AP1^71^. Data shown represent mean (SD), *n* = 3. *p < 0.05. **g** BJ-indRAS^G12V^ cells were transfected with the indicated plasmids and treated with DMSO or 4-OHT for 72 h. pGL3 constructs contain firefly luciferase reporter gene with the corresponding enhancer (none for pGL3-promoter, two different orientations for *Enh*^*AP1-OIS1*^). Relative luciferase activity is calculated by dividing the firefly luciferase activity to that of Renilla luciferase. Normalized luciferase activity is calculated by dividing the relative luciferase activity to that of pGL3-promoter for each condition. Data shown represent mean (SD), *n* = 6. *p < 0.05. **h** BJ-indRAS^G12V^ cells were transfected with the indicated enhancer constructs. Endogenous motif represents the original sequence of *Enh*^*AP1-OIS1*^, in vitro mutation construct represents mutagenesis of the AP1 consensus motif, and MU44835851 represents mutant construct bearing a C > A mutation as indicated. The cells were treated with 4-OHT for 48 h before transfection. Data shown represent mean (SD), *n* = 3. *p < 0.05

We used the *CRISPR-AP1-EnhLib* library to screen for DNA elements that are putatively regulated by AP1 and are required for the activation of OIS. BJ-indRAS^G12V^ were transduced with four independent lentiviral pools of *CRISPR-AP1-EnhLib* and selected with puromycin. Then we treated the cells with 4-OHT (RAS induction) or DMSO (control) as shown in Fig. 2a. Following four weeks of culturing, we harvested the cells, isolated genomic DNA, amplified integrated vectors by polymerase chain reaction (PCR), and used next-generation sequencing (NGS) to quantify the abundance of integrated sgRNAs present in each population. We reasoned that sgRNAs targeting REs that are required for OIS would cause bypass of senescence and sustained cell proliferation, and thus would be enriched in the cell population under oncogenic stress compared to controls (Fig. 2a). Indeed, our screen detected several sgRNAs that were highly enriched in the OIS population (Fig. 2b; Additional file 1: Table S3); among them, five showed an average enrichment fold above 1.75 over the four replicates of the screen. Notably, two of these five sgRNAs, sgRNAs-AP1^69^ and sgRNA-AP1^71^, are independent sgRNAs that target the same enhancer region (Fig. 2b), hence increasing the confidence that these are true-positive hits. ENCODE ChIP-seq data confirmed a strong binding of both FOS and JUN to this region (Additional file 2: Figure S1). Moreover, our GRO-seq data showed ~ 2-fold induction of eRNA expression from this enhancer in response to oncogenic stress. Thus, we selected this regulatory region for further validation and functional characterization and named it *Enh*^*AP1-OIS1*^.

First, using individual transductions, we validated that the introduction of sgRNAs AP1^69^ and AP1^71^ to BJ-indRAS^G12V^ cells causes a potent bypass of OIS, as judged by cell number and morphology (Fig. 2c). Second, we confirmed that introduction of these two sgRNAs to BJ-indRAS^G12V^ cells indeed results in an array of small deletions and insertions at the expected position within the AP1 binding motif in the targeted enhancer (Additional file 2: Figure S2). Third, following induction of oncogenic stress, sgRNAs AP1^69^ and AP1^71^ transduced BJ-indRAS^G12V^ cells showed a significant reduction in senescent-associated-*β*-Gal (SA- *β*-Gal) staining (Fig. 2d, Additional file 2: Figure S3) and an elevated BrdU staining (Fig. 2e, Additional file 2: Figure S3), indicative of attenuated activation of cellular senescence and of sustained cellular proliferation compared to the NT control. As expected, while the effect caused by these two sgRNAs was highly significant, it was not as strong as the effect elicited by targeting p53 (Fig. 2d and e). Last, we examined the activity of *Enh*^*AP1-OIS1*^ following the transduction of sgRNAs AP1^69^ and AP1^71^ by measuring eRNA expression at the *Enh*^*AP1-OIS1*^ locus. As expected, targeting *Enh*^*AP1-OIS1*^ by these two sgRNAs significantly compromised its activity during OIS compared to the control sgRNA (Fig. 2f).

Next, we carried out in vitro reporter assays to verify that *Enh*^*AP1-OIS1*^ functions as an enhancer and promotes the transcription of target genes. We cloned *Enh*^*AP1-OIS1*^ downstream of the Firefly luciferase gene in two orientations in the pGL3-promoter vector followed by transfection into BJ-indRAS^G12V^ cells. While we did not observe a noticeable elevation of luciferase activity in cells treated with DMSO, there was a significant increase of threefold in cells treated with 4-OHT (Fig. 2g). To verify that mutations in the AP1 binding site disrupts the enhancer activity, as we observed in sgRNA-AP1^69/71^ cells, we mutated the AP1 motif of *Enh*^*AP1-OIS1*^ and examined the effect on the enhancer activity, under the condition of oncogenic stress. Indeed, the mutations completely abolished the ability of *Enh*^*AP1-OIS1*^ to stimulate luciferase expression (Fig. 2h). In addition, we searched for tumor SMs within the AP1 binding motif (using ICGC data) and found one case in a lung cancer patient (Additional file 2: Figure S4). The mutation is a C to A substitution within the consensus motif of AP1 and located at the cut site of sgRNA-AP1^69^. To examine whether this SM disrupts the activity of *Enh*^*AP1-OIS1*^, we performed mutagenesis of *Enh*^*AP1-OIS1*^ on the reporter construct with a single nucleotide substitution. Remarkably, we observed a 50% reduction of the enhanced luciferase activity (Fig. 2h), suggesting an important role of the specified nucleotide in determining binding affinity of AP1 to this enhancer. Taken together, our functional genetic screen and subsequent focused experiments have identified and validated a novel AP1-bound enhancer whose activity is required for proper induction of OIS.

### *FoxF1* is a target gene of *Enh*^*AP1-OIS1*^

**Fig. 3:**
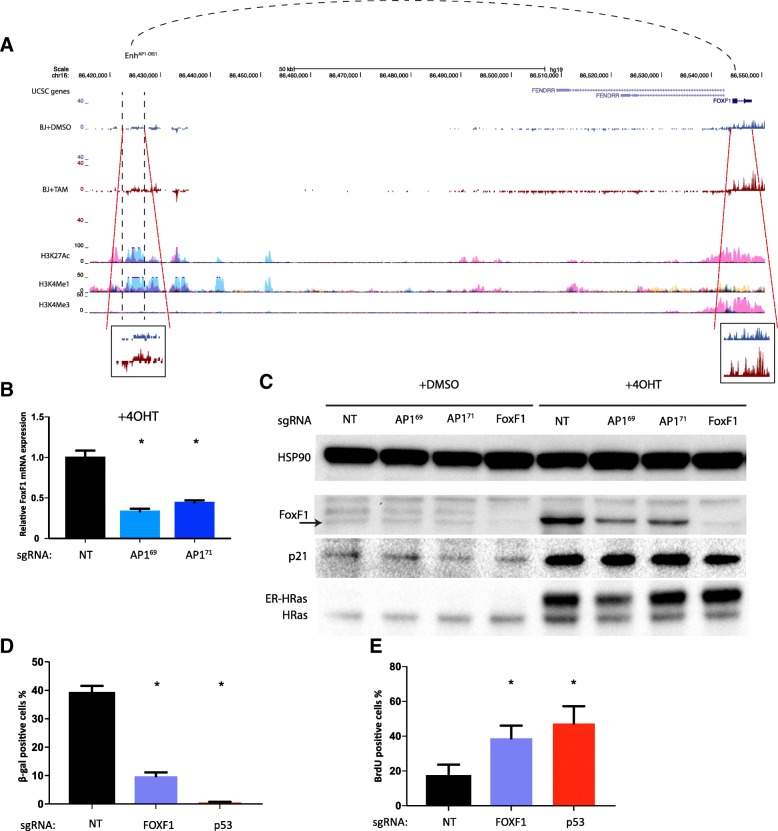
FOXF1 is the target gene regulated by *Enh*^*AP1-OIS1*^. **a** UCSC *screenshot* of GRO-seq analysis of BJ-indRAS^G12V^ cells. BJ cells were treated with DMSO or 4-OHT for 14 days. Bi-directional transcription is represented by using positive and negative values for expression in the Crick and Watson strands, respectively. The genomic regions of *Enh*^*AP1-OIS1*^ and *FOXF1* are enlarged. Note the enhancement in GRO-seq signal for both *Enh*^*AP1-OIS1*^ and *FOXF1* in BJ + 4-OHT (*brown track*) compared to BJ + DMSO (*blue track*). **b** mRNA levels of FOXF1 are reduced in sgRNA-AP1^69^ and sgRNA-AP1^71^ targeted cells under 4-OHT treatment. Data shown represent mean (SD), *n* = 3. *p < 0.05. **c** BJ-indRAS^G12V^ cells transduced with the specified sgRNAs were treated with DMSO or 4-OHT for 14 days; FOXF1, p21, and HRas protein levels were measured by western blot. HSP90 was used as the loading control. The band of FOXF1 is marked with an *arrow*. ER-HRas indicates the induced version of HRas. **d** Targeting the *FOXF1* and *p53* genes caused OIS bypass as measured by β-gal staining. Note the stronger effect of FOXF1ko compared to the effect elicited by targeting *Enh*^*AP1-OIS1*^ (Fig. 2d). Data shown represent mean (SD), *n* = 4. *p < 0.05. **e** Targeting *FOXF1* and *p53 gene* resulted in enhanced proliferation as measured by BrdU staining. Data shown represent mean (SD), *n* = 4. *p < 0.05

Next, we set up experiments to elucidate the mode of action by which *Enh*^*AP1-OIS1*^ is required for OIS. Enhancers regulate gene expression of cis-located target genes that can reside hundreds of kbp away. Examination of our GRO-seq data indicated that *FOXF1*, the nearest gene to the *Enh*^*AP1-OIS1*^ locus (located > 100 kbp downstream of it), was approximately twofold induced following oncogenic stress (Fig. 3a), suggesting a potential functional connection. No other gene in a 1-Mbp distance from *Enh*^*AP1-OIS1*^ showed such a strong effect. Furthermore, we performed RNA-sequencing (RNA-seq) with cells transduced with sgRNA-AP1^69^ and sgRNA-AP1^71^ and observed a significant reduction in the expression of FOXF1 (Additional file 2: Figure S5). We validated this result using quantitative reverse transcription PCR (qRT-PCR) analysis, which also indicated that targeting *Enh*^*AP1-OIS1*^ by either sgRNA-AP1^69^ or sgRNA-AP1^71^ results in a significant reduction in the expression level of FOXF1 under OIS conditions (Fig. 3b). Western blotting analysis confirmed this result at the protein level, in addition to confirming that FOXF1 expression is increased following oncogenic stress (Fig. 3c). Publicly available RNA Pol II ChIA-PET data (from Hela cells) indicate physical interaction between *Enh*^*AP1-OIS1*^ and the 3′ region of *FOXF1* (Additional file 2: Figure S6), which was confirmed in the chromatin conformation capture (3C) experiments of senescent BJ cells (Additional file 2: Figure S7). More importantly, the physical interactions between *Enh*^*AP1-OIS1*^ and the promoter region of *FOXF1* were significantly stronger (Additional file 2: Figure S7), suggesting a robust transcriptional regulation of *Enh*^*AP1-OIS1*^. Collectively, these results strongly point to *FOXF1* as the target gene of *Enh*^*AP1-OIS1*^.

### Loss of FOXF1 causes senescence bypass and abolishes senescence expression signatures

**Fig. 4:**
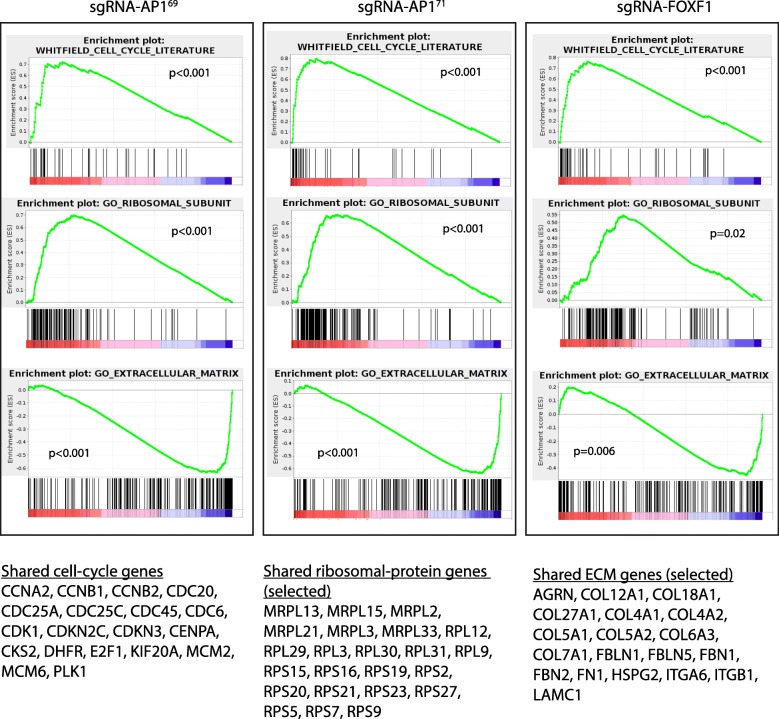
*Enh*^*AP1-OIS1*^ and *FOXF1* knockouts display expression profiles of senescence bypass. GSEA analysis of expression profiles measured in 4OHT-treated BJ-indRAS^G12V^ cells targeted by sgRNA-AP1^69^, sgRNA-AP1^71^, or sgRNA-FOXF1 compared to the profile of control 4OH-treated cells transduced with non-targeting sgRNA. A list of shared genes within each group is shown

**Fig. 5:**
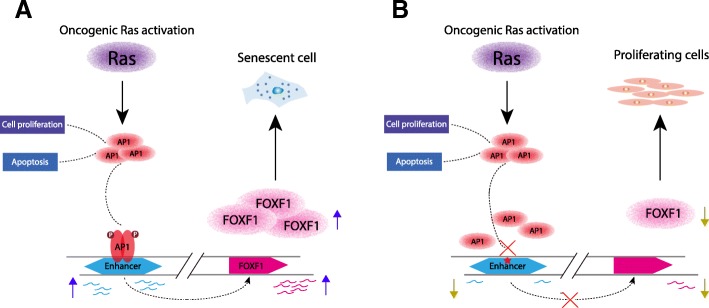
Model of *Enh*^*AP1-OIS1*^ regulation of OIS. **a** In normal BJ fibroblast cells, hyper-activation of RAS induces MAPK signaling cascade, including AP-1 TFs. Activated AP-1 TFs control different cellular functions, including cell proliferation and apoptosis. AP1 is recruited, among other enhancers, to *Enh*^*AP1-OIS1*^ and stimulates its activity. This, in turn, promotes the expression of the target gene FOXF1, diverting oncogenic signals into the pre-senescent pathway. **b** Mutagenesis of the AP-1 binding site in *Enh*^*AP1-OIS1*^ abrogates its enhancer activity and thus leads to decreased expression of FOXF1. This results in compromised induction of OIS and thus cells continue uncontrolled cell proliferation [52]

To further establish *FOXF1* as the target gene that links *Enh*^*AP1-OIS1*^ to senescence, we examined the phenotypic effect of knocking out *FOXF1*. Indeed, targeting *FOXF1* results in a strong senescence bypass phenotype, as evident by significant reduction in SA-*β*-Gal staining (Fig. 3d) and elevated BrdU staining (Fig. 3e), similar to the effect elicited by targeting *Enh*^*AP1-OIS1*^ (Fig. 2e and f). Effective *FOXF1* knockout was confirmed by western blotting analysis (Fig. 3c). Last, we used RNA-seq to globally compare expression profiles in BJ-indRAS^G12V^ cells transduced with either sgRNAs targeting *Enh*^*AP1-OIS1*^ (sgRNA-AP1^69^ or sgRNA-AP1^71^), sgRNA targeting *FOXF1*, or a control non-targeting sgRNA. Gene-set enrichment analysis (GSEA) [23] for functional characterization of the biological processes affected by these genetic manipulations showed that cell-cycle genes and genes encoding ribosomal proteins are significantly upregulated when targeting either *Enh*^*AP1-OIS1*^ or *FOXF1*, reflecting the bypass of OIS and the subsequent enhanced proliferation experienced by these cells following oncogene hyperactivity (Fig. 4). Conversely, the induction of various extracellular matrix (ECM) components that is exhibited in OIS was largely attenuated in cells with *Enh*^*AP1-OIS1*^ or *FOXF1* knockouts (Fig. 4). Taken together, our results strongly indicate that *Enh*^*AP1-OIS1*^ controls OIS through the regulation of *FOXF1* expression (Fig. 5).

## Discussion

In this study, we first found that OIS-induced enhancers are enriched for the binding motif of AP1. Based on this finding, we perform a CRISPR screen focused on AP1 motifs within enhancers that are activated upon oncogenic stress. We discovered a novel AP-1 bound enhancer, *Enh*^*AP1-OIS1*^, that is required for establishment of OIS and identified FOXF1 as the target that mediates this role. We propose a new role of AP1 in senescence via activation of FOXF1, providing an additional regulation of cell proliferation during senescence.

AP1 TFs are recruited to enhancer regions to drive oncogenic growth [24] and are broadly required for enhancer selection [25], suggesting a possible role of AP1 at enhancers. As a downstream target of RAS signaling pathway, AP1 is activated to target mitogen-responsive genes [26, 27]. Earlier studies have shown that messenger RNA (mRNA) level and activity of AP1 genes are attenuated upon entering replicative senescence [28, 29]. Altered AP1 activity is mainly due to loss of c-FOS expression and maintained JUN proteins, thus promoting JUN-JUN homodimers instead of FOS-JUN heterodimers [29, 30]. This suggests that loss of AP1 activity is possibly responsible for the irreversible growth arrest in senescent cells. Conversely, overexpression of c-FOS with increased AP1 activity is not sufficient to initiate DNA synthesis in senescent human fibroblasts [31]. Therefore, AP1 is likely not the key factor that regulates senescence, but rather a downstream factor that fine-tunes the senescence program under replicative stress (e.g. H-RAS activation). In addition, previous functional genetic screens did not indicate any of the AP1 family members as critical factors in OIS [20, 32]. Supporting this conclusion, CRISPR-mediated KOs of c-FOS and c-JUN did not result in any obvious bypass of OIS (Additional file 2: Figure S8). However, it is possible that one or few targets of AP1 mediate OIS while others antagonize it or are required for cell survival.

FOXF1 belongs to the Forkhead family of TFs. FOXA1 has been reported to promote senescence via activation of p16^INK4a^ [33] and FOXO4 inhibition induces p53 nuclear exclusion, which results in apoptosis of senescent cells [34]. The functions of FOXF1 remain to be determined, yet recent studies have implicated its role in lung regeneration by targeting genes of ECM and cell cycle progression [35], as well as promoting prostate cancer growth via the MAPK pathway [36]. To date, there has been no evidence of any connections between AP1, FOXF1, and OIS, possibly due to regulation via enhancers, rather than proximal promoters, as proposed in this study. It has been proposed that FOXF1 is a target gene of p53, which regulates cell migration and invasion [37]; and that FOXF1 is a potential oncogene, which promotes rhabdomyosarcoma by repressing p21^Cip1^ [38]. Here, we provide the first evidence suggesting that FOXF1 is a potential tumor suppressor, regulating senescence in human cells. In addition, we generated double knockout cell lines (NT + p53 ko, sgRNA-AP1^69^+ p53 ko, sgRNA-AP1^71^+ p53 ko, and FOXF1 ko + p53 ko) and we observed a strong senescence bypass phenotype (Additional file 2: Figure S9). Interestingly, we found an additive effect of proliferation in the EnhAP1-OIS1 ko and FOXF1 ko cell lines (sgRNA-AP169+ p53 ko, sgRNA-AP171+ p53 ko, and FOXF1 ko + p53 ko) compared with NT + p53 ko cells (Additional file 2: Figure S9). This suggests that FOXF1 regulates OIS in a p53 independent manner. In parallel with the canonical p53 pathway, FOXF1 regulates the expression of a subset of cell cycle and ribosomal genes (Fig. 4). Further studies should explore the exact function of FOXF1 in regulating senescence.

We propose a model in which following oncogenic induction, AP1 TFs are activated to promote cellular proliferation in response to stimuli. However, under excessive exposure to RAS, AP1 is recruited to *Enh*^*AP1-OIS1*^ to promote the expression of FOXF1 to drive cells into senescence. Disruption of the AP1 binding site within *Enh*^*AP1-OIS1*^ results in attenuated activation of FOXF1, hampering full execution of the OIS program. We attempted to generate a FOXF1 overexpression cell line while targeting *Enh*^*AP1-*OIS1^, to rescue the senescence phenotype. However, this was not successful, possibly due to the intolerance of the cells under ectopic FOXF1 expression. This result shows that although AP1 is activated by the MAPK pathway and stimulates the expression of many cell cycle genes, upon oncogenic stress it also mediates tumor suppressive effects.

Our current knowledge on cancer driver non-coding SMs is still very rudimentary, yet several studies suggested that the role of such SMs is underappreciated [39, 40]. Genome-wide analysis has revealed AP1 as a key factor at REs in cancers [41] and AP1 binding sites are frequently mutated in various cancer types [42]. Analyzing ICGC data, we found a SM within the AP1 motif of *Enh*^*AP1-OIS1*^ in a lung cancer patient and validated its functional effect by using in vitro reporter assays (Fig. 2h). This suggests a possible cancer driver effect for SMs in AP1 binding motifs. Our study further demonstrates the power of CRISPR-based screens in exploring the function of the non-coding genome.

## Conclusions

Our study provides evidence that AP1 TFs are broadly stimulated during OIS and are localized to enhancer regions to activate specific gene programs. We show that AP1 controls the senescence program via *Enh*^*AP1-OIS1*^ and its target gene *FOXF1*. We propose that AP1 is a double-edged sword in regulating cell proliferation and senescence, providing a restrictive feedback on unlimited cell proliferation.

## Methods

### Cell culture

BJ/ET/Ras^V12^and HEK293-T cells were cultured in DMEM medium (Gibco), supplemented with 1% penicillin/streptomycin (Gibco) and 10% FCS (Hyclone). To induce OIS, BJ cells were treated with 100 nM 4-OHT (Sigma) for 14 days.

### Analysis of GRO-seq data

GRO-seq was applied to control and RAS^G12V^-induced hTERT immortalized BJ cells (14 days after RAS induction). These conditions were probed using biological duplicates. Sequenced reads were aligned to the human genome (hg19) using bowtie2 [43]. Transcriptional units (TUs) were inferred from the GRO-seq data using HOMER [44]. Read counts per TU were calculated using HTseq-count [45]. A total of 76,200 TUs covered by at least 20 reads in at least one sample were detected. TU expression levels were then normalized using quantile normalization to allow comparison between samples and fold-change (FC; presented in log2 base) was calculated between the RAS-induced and control samples. To avoid inflation of high FC value for lowly expressed TUs, we set a floor value of 10 (that is, all expression levels < 10 were set to 10). Next, we defined bi-directional TUs as TUs whose start site is separated by no more than 800 bp and are transcribed on opposite strands (TU+ and TU-). As bi-directional transcription is a hallmark of transcriptional REs, we refer to these loci as REs. Overall, this analysis defined 36,497 REs. Last, a RE (bi-directional TU) was defined as OIS-induced if the expression level of both its mates was elevated by at least twofold upon RAS induction, in both duplicates. In total, 1821 OIS-induced REs were identified in our dataset (Additional file 1: Table S1).

### Motif enrichment analysis

The sequences of the OIS-induced REs were searched for statistically over-represented TF-binding motifs. We performed this de novo motif analysis using DREME [46]. For each bi-directional TU, we scanned the region between the start site of the opposite mates (TU+ and TU-) plus a margin of 200 bp to each direction. As control sequences, we extracted adjacent sequences of the same length immediately upstream and downstream from the test sequence. The binding motif of JUN/FOS was highly enriched (*p* = 1.2*10^−88^) on the OIS-induced REs. Specific occurrences of the enriched JUN/FOS motif were identified using FIMO with default parameters [47]. Overall, 762 JUN/FOS motif occurrences were found on 638 OIS-induced REs.

### CRISPR library construction and analysis

We designed a CRISPR library to target the FOS/JUN motifs in the OIS-induced REs. For 398 of the 638 OIS-induced REs with the FOS/JUN motif, we found an occurrence of the NGG PAM in a location that is expected to induce a Cas9 DNA cleavage within a margin of 5 bp with respect to the motif (that is, the cut is expected to occur within the motif or up to 5 bp from its edges). Overall, we designed 840 distinct sgRNAs, collectively targeting the FOS/JUN motif in 398 OIS-induced REs. We cloned these sgRNAs into a pLentiCRISPRv2 vector and generated a plasmid library (which we call *CRISPR-AP1-EnhLib*). Induced and control BJ-indRAS^G12V^ were transduced with four independent lentiviral pools of *CRISPR-AP1-EnhLib*. Following four weeks of culturing, we harvested library-transduced cells, isolated genomic DNA, amplified integrated vectors by PCR, and used NGS to quantify the abundance of integrated sgRNAs present in each population. Read counts were normalized to 1 M reads and enrichment ratios (FC in log2) were calculated for each sgRNA between the induced and control samples per replicate. (To avoid inflation of FC for sgRNAs covered by low number of reads, counts < 50 were set to 50). Next, average enrichment factor was calculated per sgRNA over the four replicates and was transformed to a Z score (Fig. 2b, Additional file 1: Table S3).

### Senescence-associated β-galactosidase assay

BJ cells were transduced with different sgRNA constructs and selected with puromycin. After selection, cells were seeded in triplicate in six-well plates and treated with 100 nM 4-OHT for 14 days. β-galactosidase measurement was performed by following the protocol of the Senescence β-Galactosidase Staining Kit (Cell Signaling) and at least 1000 cells were analyzed for each condition.

### BrdU proliferation assay

BJ cells were seeded in six-well plates on day 1. The next morning, cells were incubated in fresh medium for 3 h with 30 μM bromodeoxyuridine (BrdU, Sigma) followed by two washes with phosphate-buffered saline (PBS) and then fixed with 4% formaldehyde. Cells were washed twice with PBS and treated with 5 M HCl/0.5% Triton to denature DNA. Cells were neutralized with 0.1 M Na_2_B_4_O_7_. The cells were then treated with blocking buffer (3% BSA in 0.5% Tween PBS) for 30 min and incubated with anti-BrdU antibody (Dako) with blocking buffer for 2 h at room temperature. Cells were washed with PBS three times and finally incubated with FITC-conjugated anti-mouse Alexa Fluor 488 secondary antibody (Dako) in blocking buffer for 1 h, washed three times, and stained with propidium iodide for 30 min. BrdU incorporation was measured by immunofluorescence (at least 1000 cells were scored for each condition). The numbers of individual nuclei and BrdU-stained nuclei were counted using imageJ software.

### Luciferase reporter assay

The constructs with the enhancers were cloned based on pGL3-promoter (Promega) vector. The enhancer region was PCR amplified from BJ genomic DNA and inserted downstream of the firefly luciferase reporter gene. The transfection was performed by seeding 1 × 10^5^ of cultured cells in six-well plates. The next day, 500 ng of each construct (pGL3-promoter, pGL3-Enh^AP1-OIS1^-Fw, and pGL3-Enh^AP1-OIS1^-Rv) were co-transfected with 50 ng of Renilla luciferase reporter construct using Fugene-6 (Promega) following the manufacturer’s protocol. Luciferase reporter assay was performed 24 h after transfection using a Dual-Luciferase Reporter assay kit (Promega). Cells were lysed directly on the plate with passive lysis buffer for 15 min at room temperature. Firefly and Renilla luciferase activity were measured with the substrates from the kit using Centro XS3 LB960 machine (Berthold Technologies). For BJ-indRAS^G12V^, cells were pre-treated with 100 nM 4-OHT for 48 h before transfection. For HCT116, cells were treated with UV-C (50 J/m2) or MG132 (5 μM) 18 h after transfection. The luciferase assay was performed 5 h after treatment.

### Mutagenesis of *Enh*^*AP1-OIS1*^

Mutations of *Enh*^*AP1-OIS1*^ were performed using QuikChange Lightning site-directed mutagenesis kit (Agilent) according to the manufacturer’s manual. Briefly, primers for mutagenesis were designed using the online tool from Agilent. pGL3-Enh^AP1-OIS1^-Fw and pGL3-Enh^AP1-OIS1^-Rv were PCR amplified and transformed into DH5α bacteria. Single colonies from each mutant were sequence verified and used for transfection.

### RNA isolation, reverse transcription, and qRT-PCR

Total RNA was extracted using TRIsure (Bioline) reagent and following the manufacturer’s protocol. Reverse transcription was done with SuperScript III (Invitrogen) using 1 μg of total RNA per reaction. qRT-PCR was performed using a SensiFAST SYBR No-ROX Kit (Bioline) in LightCycler 480 (Roche). Primers used are listed in Additional file 1: Table S4.

### Western blot

A total of 1 × 10^6^ cells were seeded in a 10-cm dish and treated with DMSO or 4-OHT for 14 days. Cells were trypsinized and cell pellets were lysed with RIPA buffer supplemented with 1× complete protease inhibitor cocktail (Roche) following the manufacturer’s protocol. Protein concentrations were determined using a Pierce BCA protein assay kit (Thermo Scientific). Lysates were separated on SDS-PAGE gels and transferred. Membranes were immunoblotted with the following antibodies: CDKN1A (Sc-397, Santa Cruz; 1:1000); HRAS (C-20, Santa Cruz; 1:1000); FoxF1 (ab168383, Abcam, 1:1000); and HSP90 (610,418, BD Biosciences, 1:3000). Protein bands were visualized using corresponding secondary antibodies (Dako) and ECL reagent (GE Healthcare).

### Lentiviruses production and infection

HEK293T cells were seeded at the density of 5 × 10^6^ cells per 10-cm dish one day before transfection. Transfection was performed using PEI (Polyethylenimine, Polysciences) and medium was refreshed after 16 h. Virus-containing supernatant was collected 48 h after transfection by filtering through a 0.45-μm membrane (Milipore Steriflip HV/PVDF) and snap-frozen, stored at − 80 °C. BJ cells were infected and selected with the proper antibiotics 48 h after transduction for at least four days until no surviving cells remained in the no-transduction control plate.

### Chromatin conformation capture (3C) analysis

A total of 10 × 10^6^ cells were harvested in PBS for each 3C sample. Cells were centrifuged at 300 × g for 5 min at RT and resuspended in PBS/10% FBS. Cells were then incubated with an equal volume of 4% formaldehyde (2% end concentration) for 10 min and quenched with 2 M glycine solution (0.2 M end concentration), followed by centrifugation at 300 × g for 5 min at 4 °C. The cell pellet was then resuspended in PBS/10% PBS and centrifuged at 300 × g for 5 min at 4 °C. The supernatant was then discarded and snap-frozen, stored at – 80 °C. The cell pellet was lysed in 3 mL lysis buffer (50 mM Tris-HCl pH 7.5, 0.5% NP-40, 1% Triton X-100, 150 mM NaCl, 5 mM EDTA, protease inhibitor cocktail [Roche]) for 1.5 h at 4 °C, followed by centrifugation at 1000 × g for 3 min. The pellet was washed once in 1.2× restriction buffer and resuspended again in 500 μL of 1.2× restriction buffer. A total of 15 μL of 10% SDS was added to the suspension and incubated at 37 °C while shaking at 400 rpm. In total, 75 μL of 20% Triton X-100 was added to the suspension and incubated at 37 °C while shaking at 400 rpm. The samples were then centrifuged at 1000 × g for 3 min and resuspended in 500 μL of 1× restriction buffer. The digestion was performed with addition of 200 U of Csp6I (Thermo Fisher Scientific) at 37 °C overnight. The digestion efficiency was assessed the next day on agarose gel. The enzyme was then inactivated at 65 °C for 20 min and then samples were centrifuged at 1000 × g for 3 min to remove the restriction buffer. The pellet was resuspended in 7 mL of 1× ligation buffer and the ligation was performed with addition of 50 U of T4 DNA ligase at 16 °C overnight. Again, the ligation efficiency was examined on agarose gel. De-crosslinking was performed by the addition of 30 μL of protease K (Roche) at 65 °C overnight. To remove residual RNA, 15 μL of RNaseA cocktail (Ambion) was added to the samples and incubated at 37 °C for 45 min. DNA was recovered by adding 7 mL of isopropanol and 70 μL of NucleoMag 96 PCR beads (Bioke) and incubated for 30 min at room temperature. The samples were centrifuged for 3 min at 1000 × g and washed with 80% ethanol twice. Finally, the beads were dried and eluted in 300 μL of 10 mM Tris-HCl pH 7.5. To assess the physical interactions between Enh^AP1-OIS1^ and target regions, we designed a constant primer (C1) that amplifies the Enh^AP1-OIS1^ region overlapping the junction created by Csp6I enzyme. For each assessed region, we designed two primers (reverse and forward) to examine the interactions with Enh^AP1-OIS1^. The first PCR was performed with primer C1 and each candidate primer for 25 cycles. Afterwards, a nested PCR was performed using a second constant primer (C2) and each candidate primer for another 18 cycles. Finally, PCR products were resolved on 2% agarose gel. To assess the primer efficiency, we PCR amplified the genomic regions of *Enh*^*AP1-OIS1*^ and FOXF1, mixed equal molar of each fragments as the template, digested, and ligated as mentioned. Finally, the quantifications were normalized with the primer efficiencies. To examine the sequences of the PCR products, DNA bands were cut, isolated, and sanger-sequenced.

### Mutation analysis of enhancer regions

Genomic DNA of the cells transduced with sgRNAs were isolated and quantified. A total of 500 ng of the genomic DNA was used for PCR to amplify the enhancer region. We performed a two-step PCR by introducing the P5 adapter sequences in the first PCR and P7 adapters with the indexes in the second PCR. After the second PCR, the libraries were purified with CleanPCR beads (CleanNA) and quantified on 2100 Bioanalyzer using a 7500 chip (Agilent). Equimolar of each sample was taken for the final library. Libraries were sequenced using the Mi-Seq platform. Sequenced reads were aligned to the amplified enhancer region using bowtie. Bam files were analyzed to count the number of mutations (mismatches, insertions, or deletions) identified at each location in that region.

### RNA-seq library construction

Total RNA was isolated using Trisure reagent (Bioline) following the manufacturer’s protocol. Briefly, cells were lysed in Trisure, precipitated with isopropanol, and dissolved in RNase-free water. To generate strand-specific libraries, we used the TruSeq Stranded mRNA sample preparation kit (Illumina) following the manufacturer’s instructions. Briefly, 1000 ng of total RNA was polyA-enriched using oligo-dT beads and the RNA was fragmented, random primed, and reverse transcribed using SuperScript II Reverse Transcriptase (Invitrogen). Second strand complementary DNA was then synthesized, 3’-adenylated and ligated to Illumina sequencing adapters, and subsequently amplified by 12 cycles of PCR. The sequencing libraries were analyzed on a 2100 Bioanalyzer using a 7500 chip (Agilent) and pooled equimolar into a 10-nM multiplex sequencing pool.

### Sequencing

Sequencing of the CRISPR screen and RNA-seq were done using single reads of 65 bp on the Hi-Seq2500 platform (Illumina). Mutation analysis of enhancer regions was performed with single reads of 150 bp on the Mi-Seq system with a Mi-Seq reagent v2 Nano kit.

### RNA-seq analysis

Gene expression profiles were recorded in BJ-indRAS^G12V^ (14 days after RAS induction by 4-OHT treatment) transduced with CRISPR vectors that either targeted the *Enh*^*AP1-OIS1*^ using sgRNA-AP1^69^, sgRNA-AP1^71^, targeted *FOXF1* itself, or transduced with a control non-targeting sgRNA (sgRNA-NT). Sequenced reads were aligned to the human genome (hg19) using TopHat2 [48]. The number of reads mapped to each annotated gene was counted using HTseq-count [45] and then converted to RPKMs (using GENCODE v25 annotations). RPKM levels were further normalized using quantile normalization and expression levels in each sample relative to the control non-targeting sample were calculated (in log2 base). Biological pathways and processes affected by targeting the *Enh*^*AP1-OIS1*^ or *FOXF1* were sought using GSEA [23].

## Additional files

Additional file 1: **Table S1.** OIS-responsive bi-directional transcriptional units. **Table S2.** sgRNA oligo list. **Table S3.** CRISPR screen analysis. **Table S4.** Primer list. (XLSX 728 kb)

Additional file 2: **Figures S1–S9.** (PDF 13693 kb)
