# Supplementary material for: Functional CRISPR screen identifies AP1-associated enhancer regulating FOXF1 to modulate oncogene-induced senescence
Source: Genome Biol. 2018 Aug 17;19:118. doi: 10.1186/s13059-018-1494-1 (PMC6097335; doi:10.1186/s13059-018-1494-1)
Supplement: Supplementary file 2 — Figures S1–S9. (PDF 13693 kb) [file 13059_2018_1494_MOESM2_ESM.pdf]

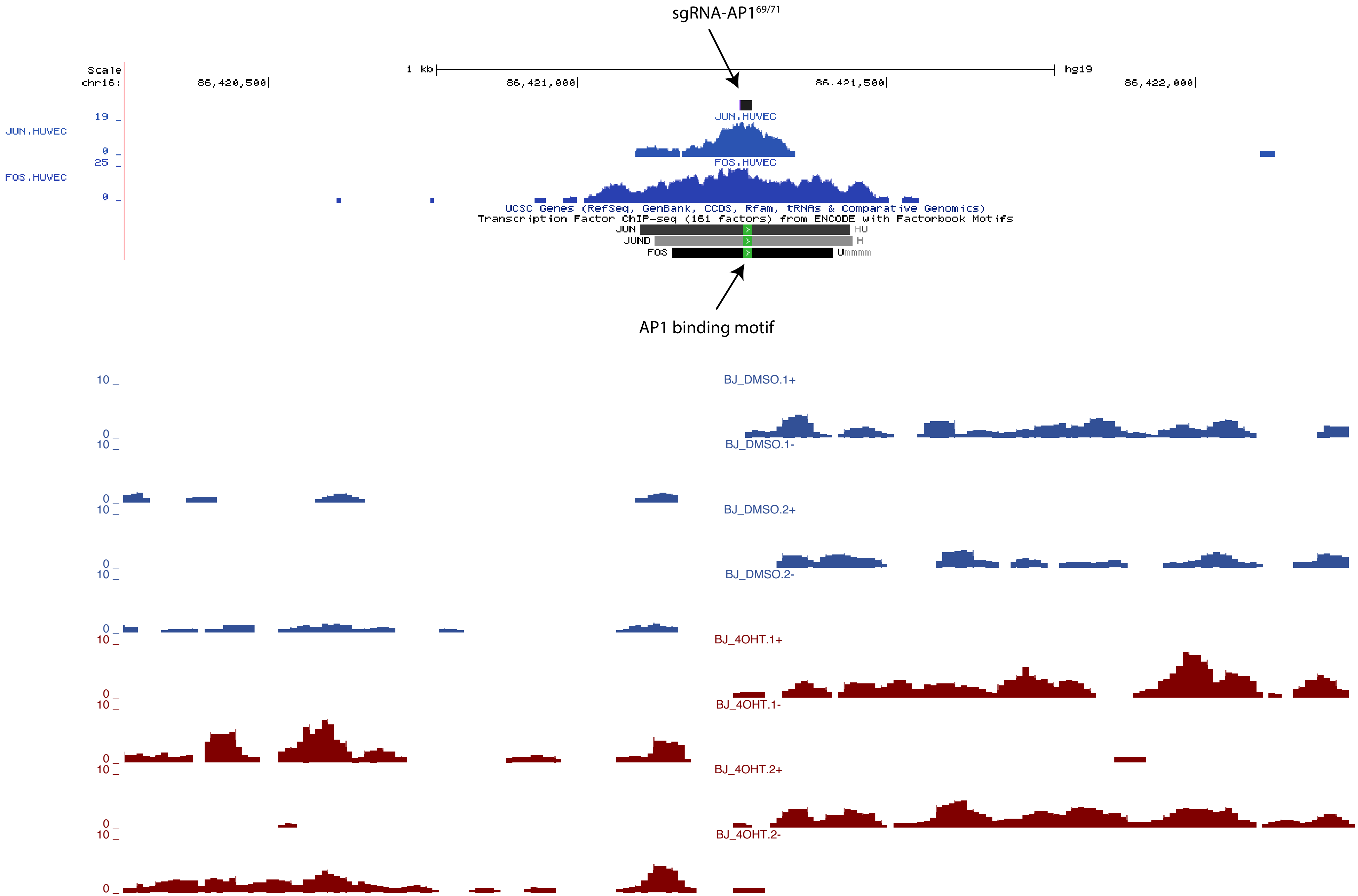

Figure S1. UCSC screenshot of EnhAP1-OIS1 with ENCODE CHIP-seq data.
ENCODE CHIP-seq data shows a significant binding of c-Fos and c-Jun to EnhAP1-OIS1. The binding of these factors coincides with the AP1 family consensus motif (Jun, JunD, and Fos in this case).

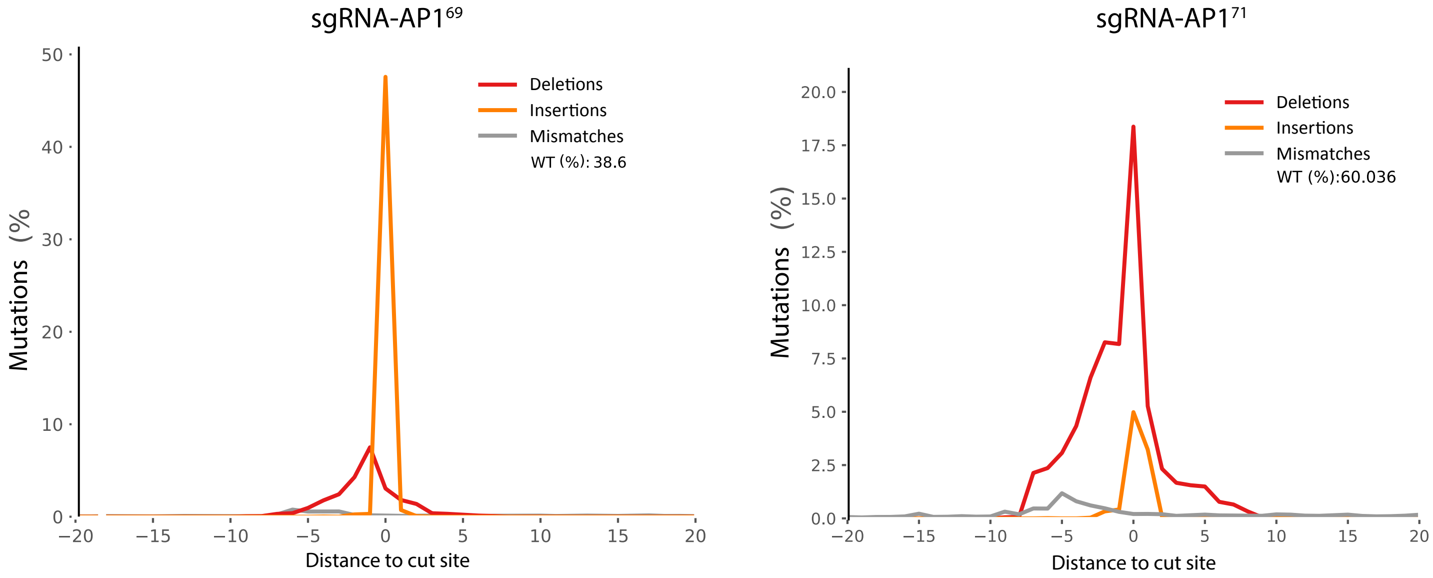

Figure S2. Mutation profiles of BJ-indRAS^{G12V} cells transduced with sgRNA-AP1^{69} and-AP1^{71}. Genomic DNA of BJ-indRAS^{G12V} cells with the indicated sgRNAs was isolated, and Enh$^{AP1\text{-}OIS1}$ region was PCR amplified, subjected to deep sequencing and analysed for mutations. Proportion of wild-type or mutated (by mismatch, deletion or insertion) base calls is indicated as a function of its distance from the Cas9 cleavage site. For sgRNA-AP169, the majority of the mutations are single nucleotide insertions, while for sgRNA-AP1^{71} deletions around the cleavage site were more prevalent.

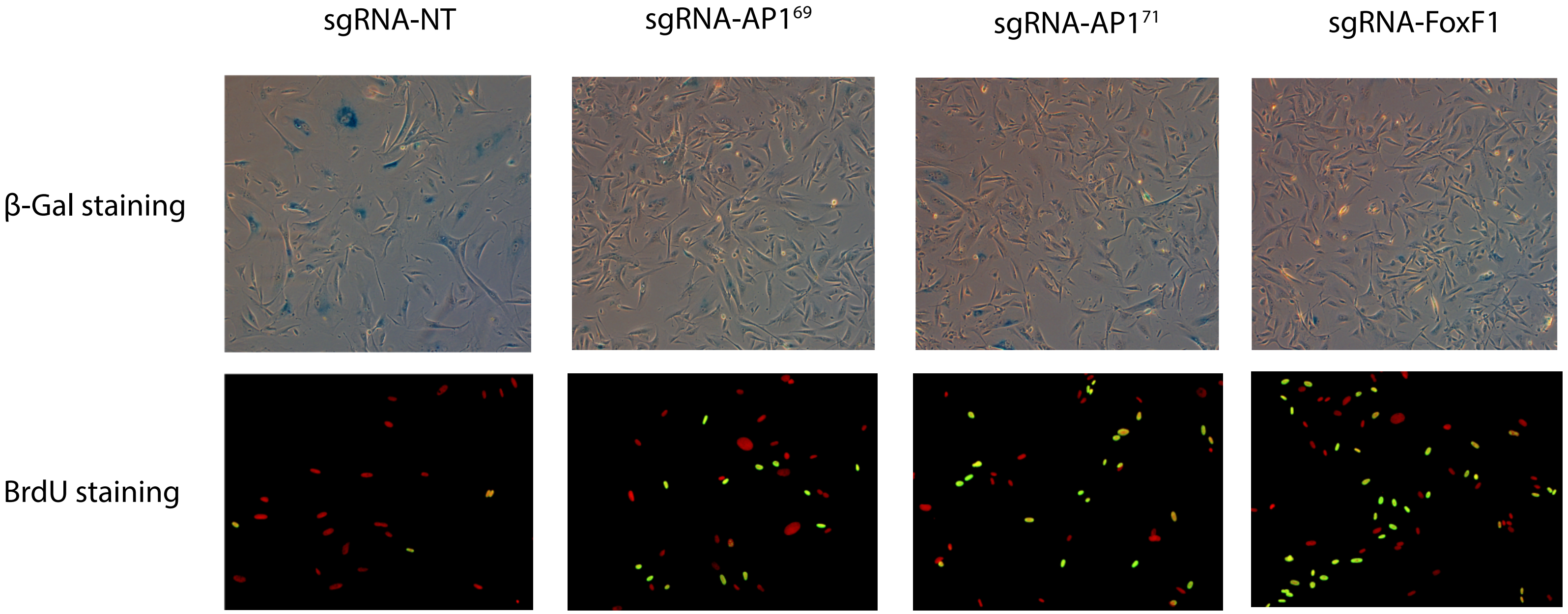

Figure S3. Representative pictures of β-Gal assay and BrdU staining.
Representative pictures of β-Gal assay and BrdU staining experiments used for the analysis of the results shown in Fig. 2-3. Each column represents the cells infected with the indicated sgRNA. The cells were treated with 4-OHT for 14 days prior analysis. Upper panel shows the β-Gal staining experiments with senescent cells stained as blue. Lower panel shows the BrdU staining experiments with cells incorporated with BrdU stained as green and nucleus stained as red.

Summary

ID	MU44835851
DNA change	chr16:g.86421280C>A
Type	single base substitution
Reference genome assembly	GRCh37
Allele in the reference assembly	C
Functional Impact	Unknown

Project	Site	Tumour Type	Tumour Subtype	# Donors affected ▾
LUSC-KR	Lung	Lung cancer	Adenocarcinoma, Squamous cell carcinoma	1 / 66 *(1.52%)*

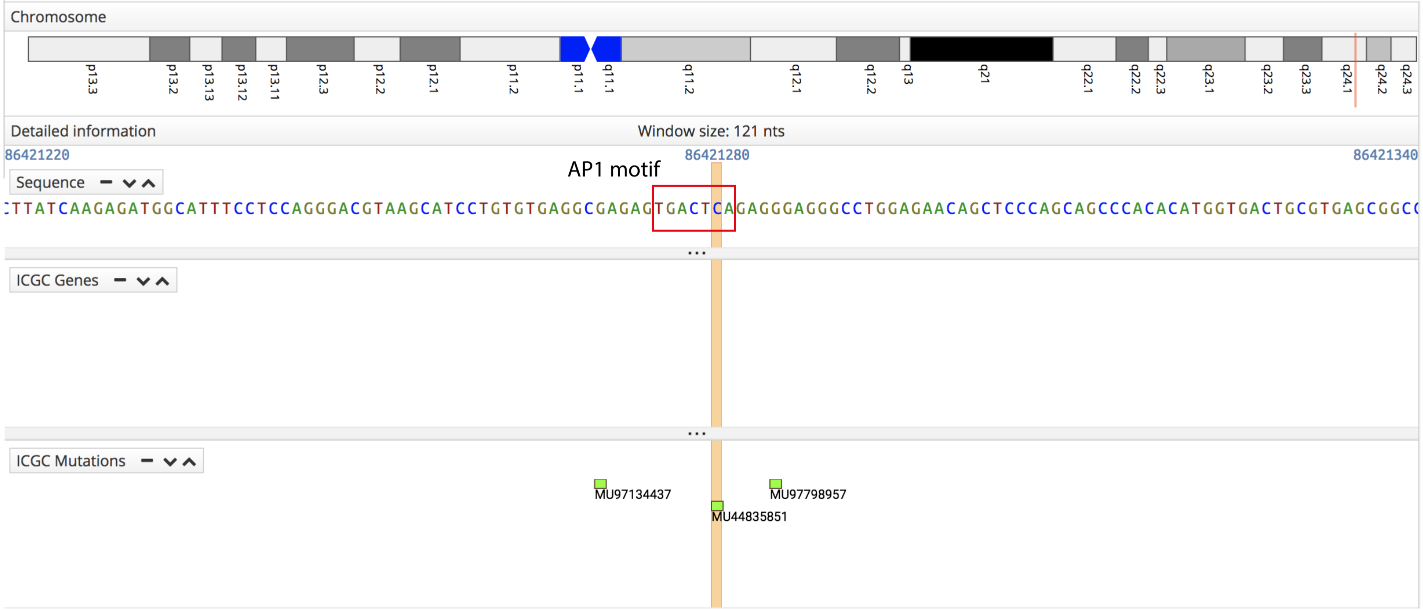

Figure S4. Somatic mutation in the AP1 motif within Enh$^{AP1\text{-}OIS1}$.
Screenshots from ICGC portal show a single nucleotide substitution within the AP1 motif in Enh$^{AP1\text{-}OIS1}$ detected in a patient with lung cancer.

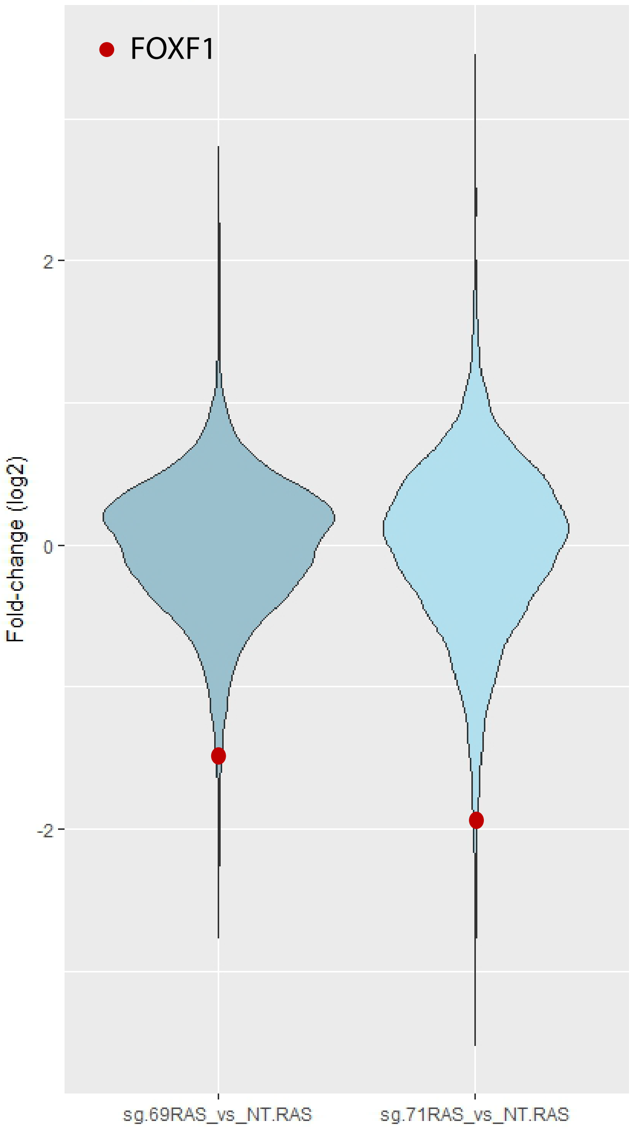

Figure S5. RNA-seq analysis indicates FOXF1 as a target gene of $Enh^{AP1-OIS1}$. Gene expression levels were measured in BJ cells targeted by either $sgRNA-AP1^{69}$, $sgRNA-AP1^{71}$ or sgRNA-NT negative control. Violin plots show the distribution of fold change of gene expression (in log2 base) calculated for the comparison between the $sgRNA-AP1^{69}$ and $sgRNA-AP1^{71}$ samples and the sgRNA-NT control. FOXF1 is marked by a rod dot. Its expression was markedly decreased by targeting $Enh^{AP1-OIS1}$ by either $sgRNA-AP1^{69}$ or $sgRNA-AP1^{71}$.

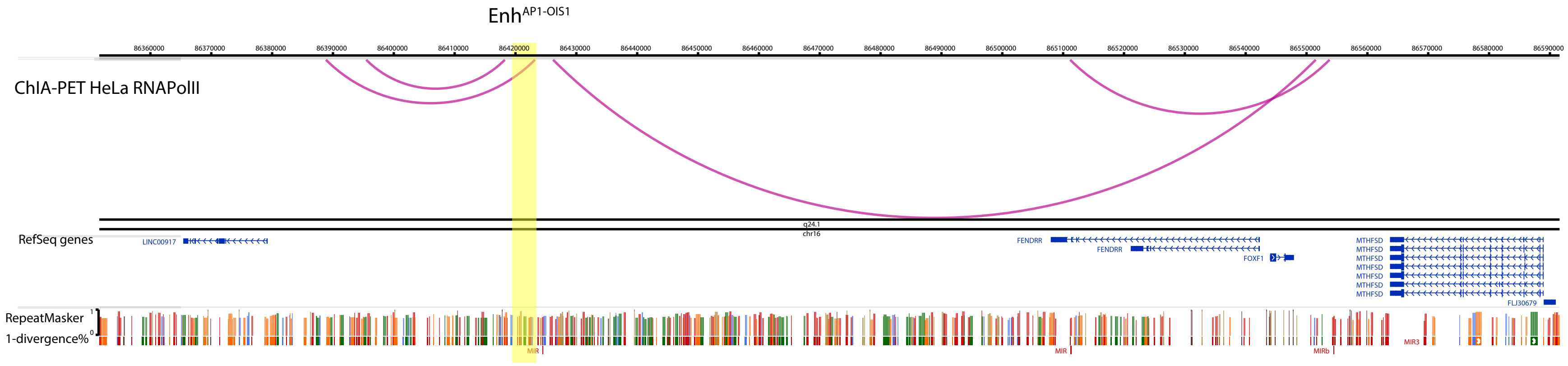

Figure S6. RNA PolII ChIA-PET data support direct interaction between Enh$^{AP1\text{-}OIS1}$ and FOXF1.
Each CHIA-PET experiment is indicated with the name of cell lines and the antibody used for the pull down on the left. Enh$^{AP1\text{-}OIS1}$ is highlighted in yellow and inferred physical interactions between two regions are presented by loops. Data is taken from the WashU epigenome browser [52].

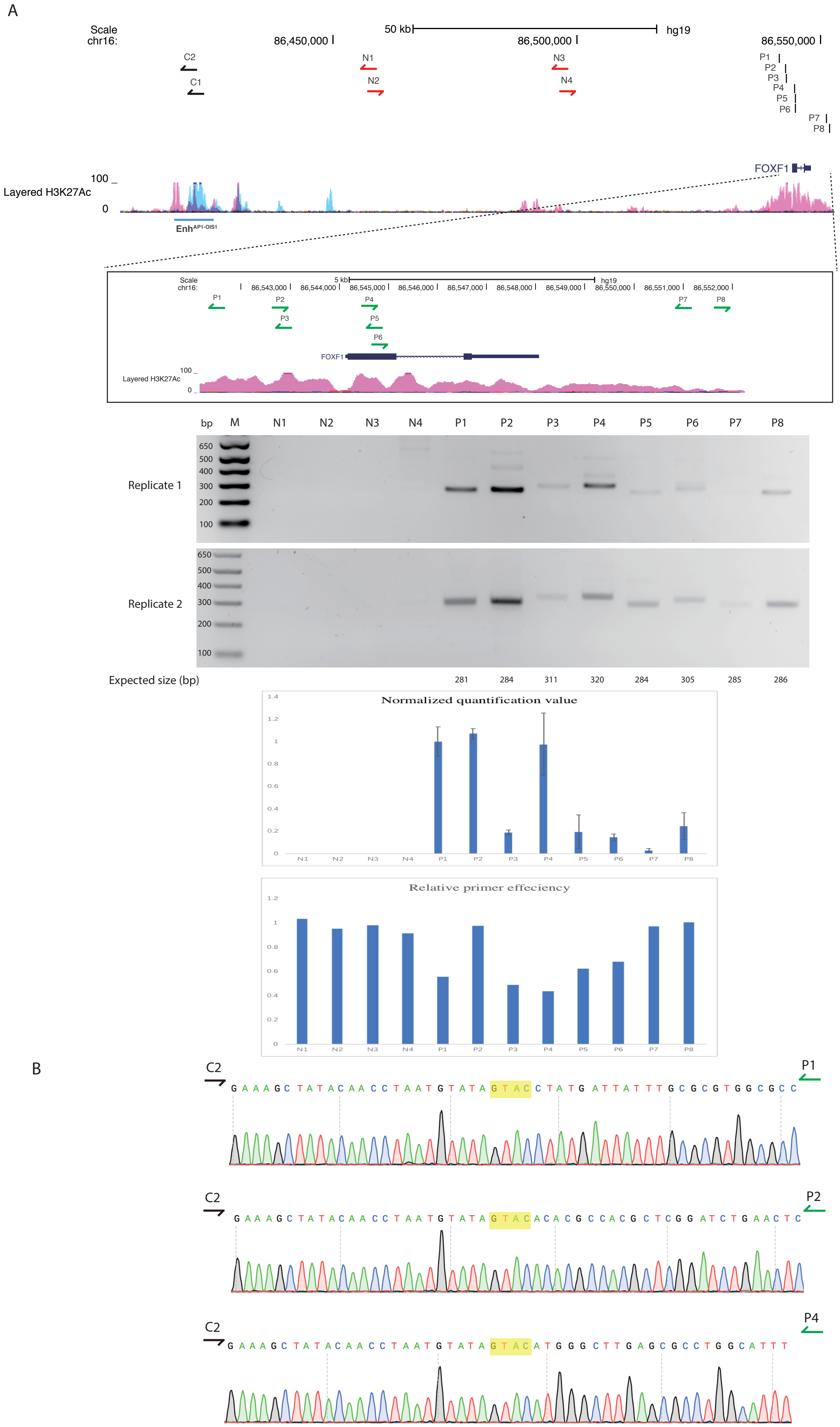

Figure S7. 3C experiment reveal direct interaction between Enh$^{AP1\text{-}OIS1}$ and FOXF1.
A. Genome browser presentation of the location of each primer used in 3C analysis. Constant primers (C1, C2) used to amplify Enh$^{AP1\text{-}OIS1}$ are indicated in black arrows. Negative control regions with no interaction with Enh$^{AP1\text{-}OIS1}$ are amplified with primers indicated in red arrows (N1-N4). FOXF1 regions with potential interactions with Enh$^{AP1\text{-}OIS1}$ are amplified with primers indicated in green arrows (P1-P8). Agarose gel images from two independent biological replicates are shown with the expected sizes of the PCR products. The quantification of the gels was performed by normalizing to the primer efficiencies. Values shown are further normalized to the quantification value of P1. B. Sanger sequencing results from the indicated PCR products. Csp6I restriction sites are highlighted in yellow. The PCR were performed using the indicated primer pairs.

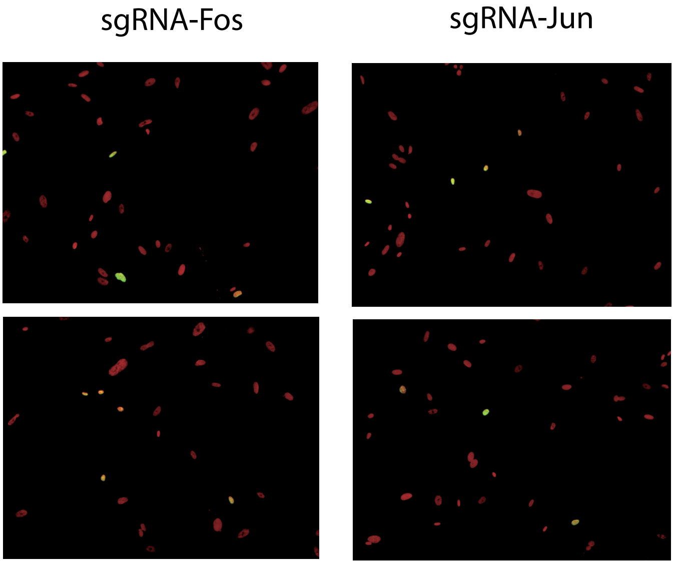

Figure S8. Disruption of Fos and Jun could not bypass senescence. Representative pictures of BrdU staining experiments with sgRNAs against c-Fos and c-Jun genes. BJ cells were treated with 4-OHT for 14 days. Each column shows two pictures from the same experiment.

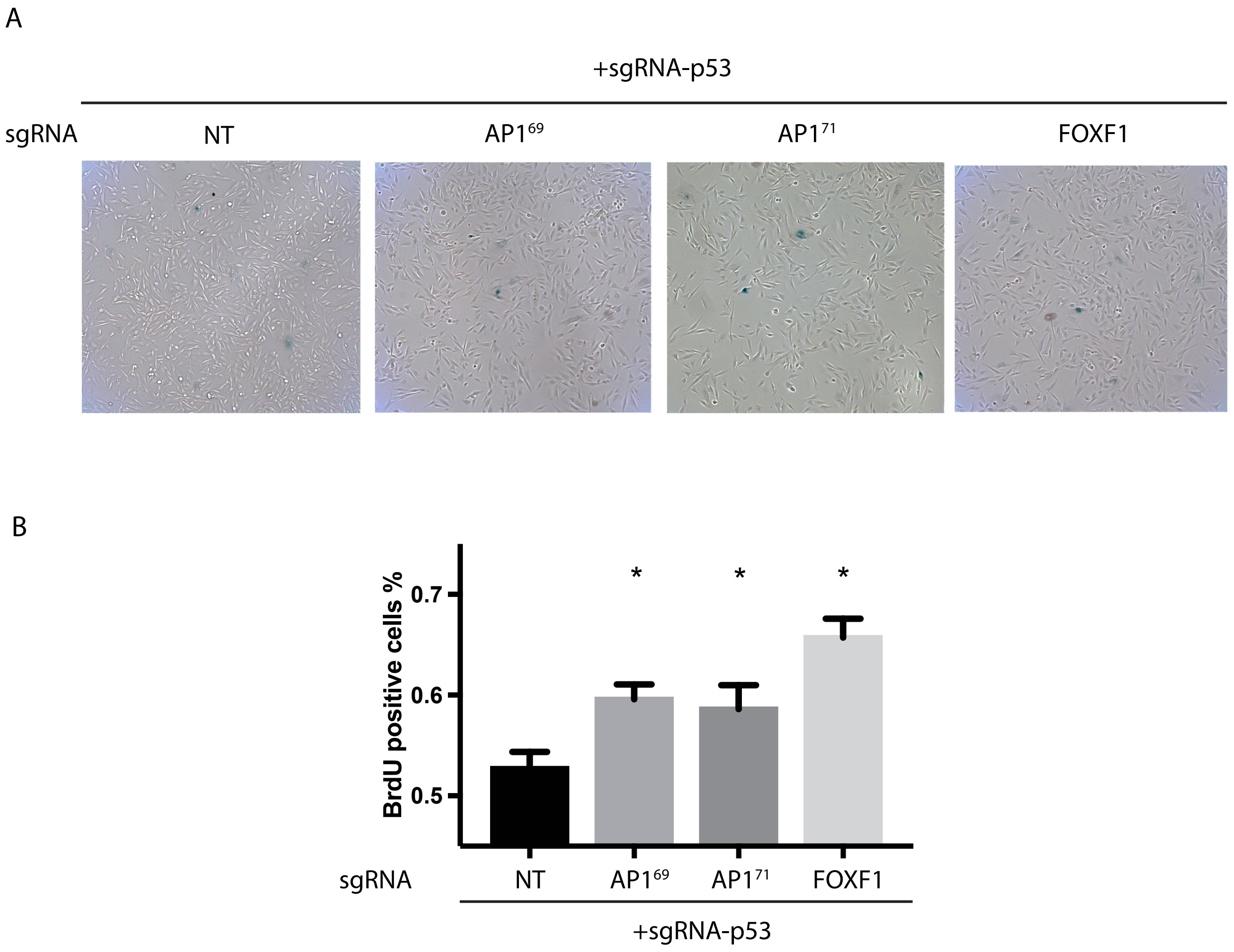

Figure S9. FOXF1 function is independent of p53 during OIS.
A. Representative images of the SA-β-gal staining of cells under the indicated conditions and after 14 days 4-OHT treatment. B. The proliferation levels of the various sgRNA-transduced BJ-in-dRAS^{G12V} cells (indicated in the figures) was quantified using BrdU assay, (* $p < 0.05$, two-tailed Student's t-test). For every condition, the percentage of BrdU-positive cells was normalized to control cells (NT+p53 ko).
